# Pharmacokinetic Study and Tissue Distribution of Lorlatinib in Mouse Serum and Tissue Samples by Liquid Chromatography-Mass Spectrometry

**DOI:** 10.1155/2019/7574369

**Published:** 2019-03-04

**Authors:** Wei Chen, Yafei Shi, Shuya Qi, Haiyan Zhou, Chunyu Li, Dujia Jin, Guohui Li

**Affiliations:** ^1^Department of Pharmacy, National Cancer Center/National Clinical Research Center for Cancer/Cancer Hospital, Chinese Academy of Medical Sciences and Peking Union Medical College, Beijing 100021, China; ^2^Institute of Materia Medica, Chinese Academy of Medical Sciences and Peking Union Medical College, Beijing 100050, China

## Abstract

In the present study, we developed and validated a rapid and simple liquid chromatography-tandem mass spectrometry (LC-MS/MS) method for the determination of lorlatinib in mouse serum and tissue samples, and such a method was successfully applied to investigate the pharmacokinetic study and tissue distribution of lorlatinib after oral administration. Samples were processed with methanol to precipitate protein and extract drugs, and Afatinib-d6 was used as the internal standard (IS). For LC-MS/MS analysis, compounds were separated on a C18 column by gradient elution (0.1% of formic acid and methanol) at 0.5 mL/min in the positive-ion mode with *m*/*z* 407.28 [M + H]^+^ for lorlatinib and *m*/*z* 492.10 [M + H]^+^ for IS. Good linearity was observed within the calibration ranges. Selectivity, accuracy (−6.42% to 8.84%), precision (1.69% to 10.98%), recoveries (91.4% to 115.0%), and matrix effect (84.2% to 110.6%) were all within the acceptable ranges. After oral administration, serum concentration of lorlatinib quickly achieved the maximal concentration (2,705.683 ± 539.779 *μ*g/L) at 0.625 ± 0.231 h. The highest concentration was detected in the liver (3,153.93 ng/100 mg), followed by the stomach (2,159.92 ng/100 mg) and the kidney (548.83 ng/100 mg). In conclusion, a simple and rapid detection method was established and validated for determination of lorlatinib in blood and tissue samples of mouse. The pharmacokinetic study and tissue distribution of lorlatinib were successfully investigated using this method.

## 1. Introduction

Crizotinib, a first-generation anaplastic lymphoma kinase (ALK) inhibitor, and ceritinib and alectinib, the second-generation ALK inhibitors, have been used as the first-line treatment for non-small-cell lung cancer (NSCLC) carrying ALK or ROS1 rearrangements [1]. However, there are two important clinical issues in ALK-positive NSCLC treatment using ALK inhibitors. Brain metastases are commonly detected in ALK-rearranged NSCLC [2], and the cumulative incidence of BM is 58.4% at 3 years [3]. Preventing BM is the first challenge in ALK-positive NSCLC treatment. The first-generation ALK-tyrosine kinase inhibitors (TKIs) have poor central nervous system penetration, and the intracranial efficacy of second-generation ALK-TKIs have been improved [4]. Besides, the second challenge is that several resistance mechanisms, such as secondary mutation resistance, and activation of bypass signaling pathways may cause drug resistance during tumor treatment [5]. To control intracranial disease and mutation resistance, lorlatinib is developed as a third-generation TKI that targets ALK and ROS1 [1]. Moreover, *in vitro* assays exhibit that lorlatinib has greater potency and selectivity than previous generations of ALK-TKIs [6, 7]. More importantly, it remains highly active in patients who have been treated with previous generations of ALK-TKIs [8]. In addition, lorlatinib can induce intracranial responses and cause neurological side-effects due to its high brain penetration ability [8].

Few bioanalytical assays have been described for the quantification of lorlatinib in mouse serum. In previous studies, protein precipitation is used as a pretreatment step and liquid chromatography-tandem mass spectrometry (LC-MS/MS) determination is applied to quantify lorlatinib [9–11]. However, there is no method available for quantification of lorlatinib in tissues containing more endogenous substances. The distribution of lorlatinib in the brain is crucial for controlling brain metastases. Nevertheless, there are no *in vivo* studies available about the biodistribution of lorlatinib. Therefore, it is crucially necessary to establish a simple and sensitive method for quantification of lorlatinib in serum and tissue samples.

In the present study, we developed a simple and sensitive LC-MS/MS method for determination of lorlatinib in mouse serum and tissue samples.

## 2. Materials and Methods

### 2.1. Chemicals and Reagents

Lorlatinib (>99.9%) was obtained from MedChem Express (USA), and Afatinib-d6 (>99.2%) was purchased from Toronto Research Chemicals Inc. (Canada). Methanol and acetonitrile of HPLC-grade were supplied by Merck Co. (Germany). Water for preparing chromatographic eluents was provided by Guangzhou Watsons Food & Beverage Co., Ltd. (China). Water applied for other experiments was purified by reverse osmosis. All of other reagents were of analytical grade unless otherwise indicated.

### 2.2. Equipment

The LC-MS/MS system was composed of the chromatographic system, consisting of two Accela pumps (ACQUITY UPLC I-CLASS BSM), an autosampler (ACQUITY UPLC I-CLASS SM-FIN) and a column oven (ACQUITY UPLC I-CLASS CH-A), and a Xevo TQ-S mass spectrometer equipped with heated electrospray ionization (Waters, USA). The LC-MS/MS was performed using the MassLynx software (version 4.1). Briefly, 2 *μ*L pretreated sample was injected into an Aquity UPLC® BEH C18 column (2.1 mm × 50 mm, dp = 1.7 *μ*m, Waters, USA), which was protected by an In-line Filter Assembly (ACQUITY GUARD FILTER, USA). H1650R desktop high-speed refrigerated centrifuge was purchased from Shanghai Lu Xiang Yi Centrifuge Instrument Co., Ltd. (China). BT125D electronic balance was provided by Sartorius Lab Instruments GmbH & Co. KG (Germany). The G560E vortex mixer was obtained from Scientific Industries (USA). The XINW-M48 high-throughput tissue homogenizer was supplied from Shanghai Xin Weng Scientific Instrument Co., Ltd. (China).

### 2.3. HPLC-MS Conditions

The mobile phase consisted of 0.1% formic acid solution (A) and methanol (B), and the flow rate of the mobile phase was set at 0.5 mL/min. The column oven was maintained at 50°C. The gradient elution program was set as follows: 0–0.5 min: 20% B, 0.5–1.7 min: 20–45% B, 1.7–1.8 min: 45%–100% B, 1.8–2.1 min: 100% B, 2.1–2.2 min: 100%–20% B, 2.2–2.5 min: 20% B.

**Figure 1:**
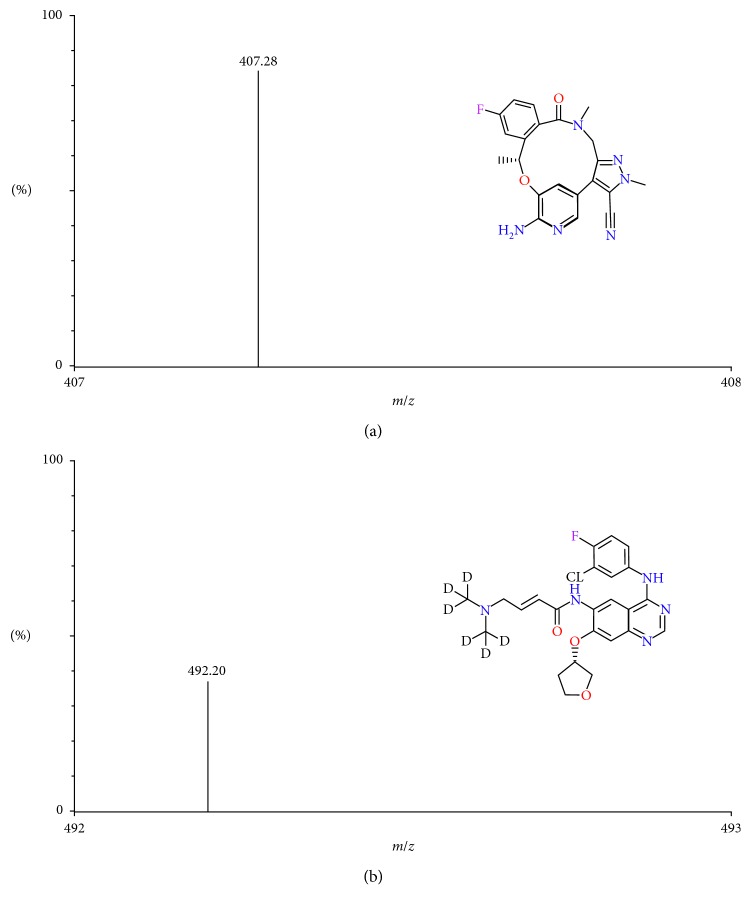
Chemical structures and typical mass spectra of lorlatinib (a) and IS (b) in the positive mode.

The MS parameters were set as follows: electron spray ionization (ESI) source, positive-ion mode; capillary voltage: 3,000 V; desolvation gas flow: 650 L/h; desolvation temperature: 350°C; cone gas flow: 150 L/h; detection mode: multiple reaction monitoring. The MS parameters of lorlatinib and internal standard (IS) are shown in Table 1. The structures and typical mass spectra of lorlatinib and IS in the positive-ion mode are illustrated in Figure 1.

### 2.4. Animals

All the animal-related experiments were performed in accordance with guidelines of Institutional Experimental Animal Ethical Committee. Kun Ming mice (18–20 g) were obtained from the Beijing HFK Bioscience Co., Ltd. (Beijing, China). All mice were given free access to normal diet and water during the experiment with an exception that mice were fasted for 12 h prior to drug administration.

### 2.5. Preparation of Calibration Standards and Quality Control (QC) Samples

Lorlatinib was dissolved in methanol to obtain a primary stock solution of 1.0 mg/mL and kept at 4°C prior to further analysis. Working standard solutions were prepared by diluting the primary stock solution with methanol to a final concentration of 100.0 *μ*g/mL. Serum and tissue calibration standards were prepared by adding working standard solutions to blank mouse serum and methanol extraction of blank tissue homogenate. Tested tissues included the skeletal muscle, fat, cerebrum, heart, liver, spleen, lung, kidney, stomach, small intestine, and large intestine. Eight concentrations of serum calibration standards were 1,000, 500, 200, 100, 50, 20, 10, and 5 ng/mL. Seven concentrations of tissue calibration standards were 500, 200, 100, 50, 20, 10, and 5 ng/mL. Serum and tissue QC samples were produced in the same way as abovementioned. Four concentrations of serum QC samples were 800 (QCH), 100 (QCM), 10 (QCL), and 5 (LLOQ) ng/mL. Four concentrations of tissue QC samples were 400 (QCH), 100 (QCM), 10 (QCL), and 5 (LLOQ) ng/mL. Calibration standards and QC samples were stored at −20°C prior to further analysis.

### 2.6. Sample Pretreatment

#### 2.6.1. Serum Sample Pretreatment

In order to make all serum samples fall within the range suitable for testing, 10 *μ*L of serum sample was diluted to 100 *μ*L with blank serum. The diluted serum sample was evenly mixed using vortex-mixing. A mixture sample was prepared by adding 200 *μ*L of IS working solution (10 ng/mL) to 20 *μ*L of diluted serum sample, and the mixture was vortex-mixed for 3 min. In order to extract lorlatinib, the supernatant was obtained by centrifugation (11,000 rpm, 15 min, 4°C). Then, 100 *μ*L of supernatant was diluted to 200 *μ*L with purified water, and the mixture was immediately vortex-mixed for 3 min.

#### 2.6.2. Tissue Sample Pretreatment

Tissue samples were weighed, and methanol was added to tissue samples at a ratio of methanol: tissue = 2 mL: 100 mg. Subsequently, tissue samples were homogenized using a high-throughput tissue homogenizer and vortex-mixed for 3 min. Supernatant was obtained by centrifuging at 11,000 rpm for 15 min. Then, 400 *μ*L of 40 ng/mL IS solution was added to 100 *μ*L supernatant. After vortex-mixing, supernatant was isolated by centrifugation.

### 2.7. Method Validation

In terms of selectivity, linearity, precision and accuracy, matrix effect, and extraction recovery, the LC-MS/MS method for lorlatinib quantification was validated, procedures referring to FDA guideline for Industry, Bioanalytical Method Validation [12].

#### 2.7.1. Selectivity

The selectivity was testified by comparative analysis of blank samples (without lorlatinib or IS), spiked blank samples, and samples after lorlatinib administration.

#### 2.7.2. Calibration

For blood sample and each tissue sample, the *x*-*y* scatter plot consisting of eight different points was drawn in accordance with the quantification of calibration standards. In this plot, the *y*-axis measured the ratio of the analyte and IS and *x*-axis determined the lorlatinib concentration. The linear calibration curve was calculated by the regression model based on the *x*-*y* plot. For the nominal value, the total allowable accuracy and precision were within 15%. For the lower limit of quantitation (LLOQ), the allowable accuracy and precision were within 20%.

#### 2.7.3. Accuracy and Precision

Accuracy refers to how close the detection value was to true value, and it was described by relative error (RE%). Precision refers to the magnitude of random errors of measurements, and it was expressed by relative standard deviation (RSD%). The within-day accuracy and precision were expressed by analyzing the data set obtained from replicated QC samples. The between-day accuracy and precision were calculated by the data set obtained from repeated experiments (including the production, pretreatment, and quantification of the QC samples) during 3 consecutive days. The acceptable within-day and between-day accuracy and precision should not exceed 15%, while they were less than or equal to 20% for LLOQ.

#### 2.7.4. Recovery and Matrix Effect

The extraction recovery was expressed at high, medium, and low QC levels by calculating the corrected peak area ratio of blank samples spiked with lorlatinib before extraction to blank samples spiked with lorlatinib after extraction. The matrix effect (serum and tissue homogenates) was assessed by the postextraction spike method. Matrix effect was described by the corrected peak area ratio of blank samples spiked with lorlatinib after extraction to the solution of lorlatinib at equivalent concentration prepared in mobile phase.

### 2.8. Pharmacokinetic Study and Tissue Distribution

The mice were orally administered with 10 mg/kg lorlatinib. Blood and tissue samples were collected from mice at 0.5, 1, 2, 4, 8, and 24 h after administration. The blood samples were transferred into glass containers and clotted at room temperature for 1 h. Once the clot was formed, blood sample was centrifuged at 4,000 rpm for 10 min, and supernatant was collected. The serum was transferred into another tube and stored at −80°C prior to further analysis. Tissue samples were rinsed by physiological saline, and then excess physiological saline was blotted up. The tissue samples were transferred into PP plastic bags and stored at −80°C until further detection.

### 2.9. Data Analysis

The pharmacokinetic parameters of lorlatinib were estimated with noncompartment model by DAS (Data Analysis System) software (version 3.0, BioGuider Co., Shanghai, China). Moment analysis was used as a noncompartmental approach to obtain various pharmacokinetic parameters. Noncompartmental approach allowed the estimation of pharmacokinetic parameters without assuming any structural properties of the pharmacokinetic behavior of lorlatinib. The area under the curve (AUC) and the first moment curve (AUMC) were calculated by using the linear trapezoidal method, which is required by FDA. The half-life (t1/2) was estimated as MRT multiplied by 0.693. *C*_max_ was defined as maximal concentration, and *T*_max_ was defined as the time at which the *C*_max_ was observed. The tissue distribution was described by quantifying lorlatinib in various tissues at 0.5, 1, 2, 4, 8, and 24 h after administration. All data were summarized to calculate means and standard deviations, and then the concentration-time curve was plotted.

## 3. Results and Discussion

### 3.1. Conditions for MS and HPLC

Conditions for MS and HPLC initially were referred to existing literature for lorlatinib [9]. In order to determine the lorlatinib concentration in tissues, chromatographic and mass spectrometric conditions were optimized according to multiple considerations, such as retention time, ionization behavior of IS, and proteins and metabolites in tissue samples. Considering the accuracy and precision of analysis in the pilot experiments, Afatinib-d6 was selected as the IS for further method validation and drug quantification. The positive-ion mode was chosen at *m/z* 407.28 and *m/z* 492.10 for lorlatinib and IS, respectively.

### 3.2. Validation

#### 3.2.1. Selectivity

**Figure 2:**
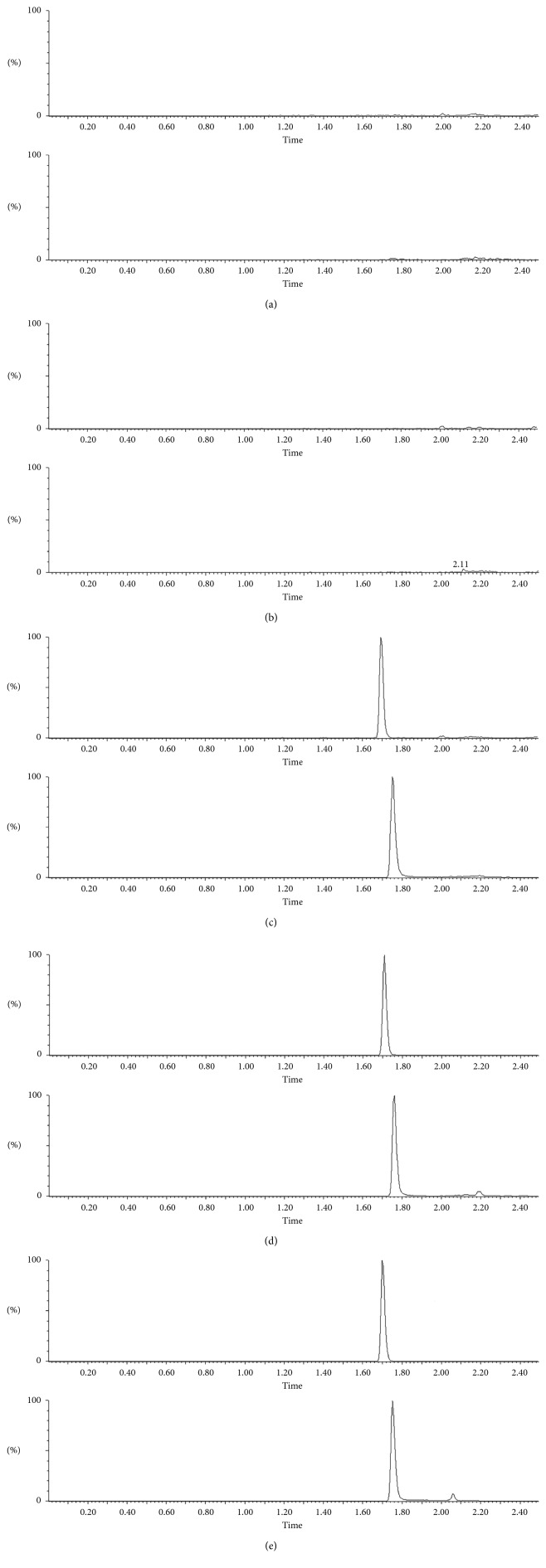
Chromatograms of serum and tissues: (a) blank serum, (b) blank lung, (c) blank serum spiked with lorlatinib and IS, (d) serum sample obtained at 30 min after oral administration of lorlatinib, and (e) lung sample obtained at 30 min after oral administration of lorlatinib. 1: lorlatinib; 2: IS.

No interfering peaks were shown in the analysis of blank blood samples (*n*=6) and tissue samples (*n*=6).  Figure 2 illustrates representative chromatograms of blank samples, blank samples spiked with lorlatinib and IS, and samples after lorlatinib administration. Response of blank samples was compared with that of the LLOQ samples. The signal noise of lorlatinib was less than 20% of LLOQ response level of lorlatinib. The signal noise of IS was less than 1% of the LLOQ response level of IS. Accordingly, the analyte and IS would not be interfered by endogenous components while using the newly developed method for analysis.

#### 3.2.2. Linearity

For blood sample and each tissue sample, the linear calibration curve was calculated by linear regression model.  Table 2 presents the linear equation of calibration curves, as well as its linear regression coefficient and linear ranges. Linearity of all calibration curves over the concentration ranges met the requirements (*R*^2^ > 0.99) of the pharmacokinetic study. All LLOQs were below the minimum concentration required for analysis, demonstrating the allowance of lorlatinib quantitation at 24 h after administration.

#### 3.2.3. Accuracy and Precision

Table 3 shows the accuracy and precision for blood and tissue samples. All within-day accuracy and between-day accuracy at three different concentrations ranged from −6.42% to 8.84%, and precision ranged from 1.69% to 10.98%, indicating that the precision and accuracy of the HPLC–MS/MS analysis method met the acceptable criteria [12].

#### 3.2.4. Recovery and Matrix Effect

All recoveries were within the range of 91.4%–115.0%, suggesting no significant extraction losses in the pretreatment of samples. The matrix effect of lorlatinib ranged from 84.2% to 110.6%. The results of matrix effect study demonstrated that no significant response could be observed at the retention time of lorlatinib.  Table 4 shows the recovery and matrix effect.

### 3.3. Pharmacokinetic Study and Tissue Distribution

**Figure 3:**
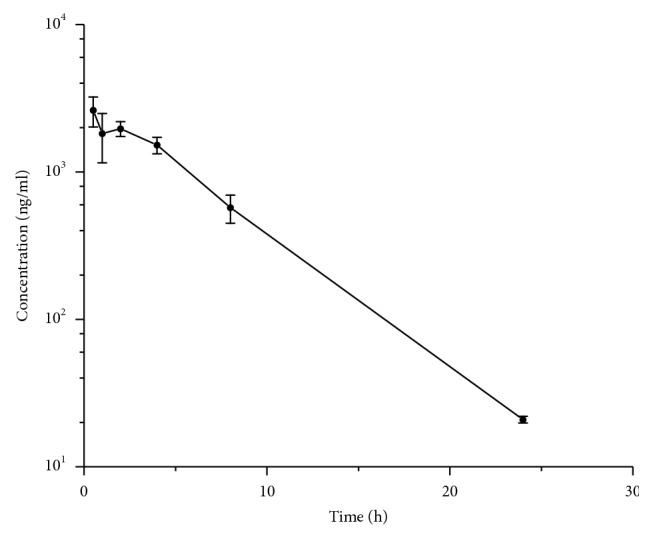
Mean serum concentration-time curves of lorlatinib in serum after oral administration at a dose of 10 mg/kg in mice (*n*=8).

Noncompartmental models were used to describe pharmacokinetics of lorlatinib.  Figure 3 shows the mean serum concentration versus time curve.  Table 5 lists the major pharmacokinetic parameters calculated by the noncompartmental model. After oral administration, blood concentration of lorlatinib quickly achieved the maximal concentration (2,705.683 ± 539.779 *μ*g/L) at 0.625 ± 0.231 h. After 24 h, the lorlatinib concentration in serum was 20.92 ± 1.07 *μ*g/L, showing rapid clearance of lorlatinib from the body.

**Figure 4:**
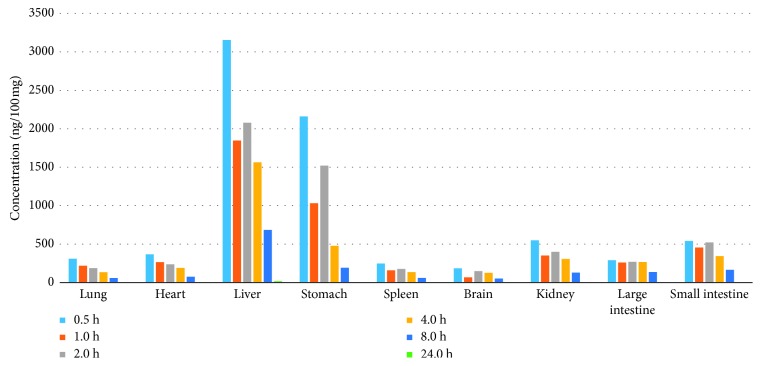
Tissue distribution profile of lorlatinib in tissues after oral administration at a dose of 10 mg/kg in mice.

To illustrate the lorlatinib distribution, we analyzed the lorlatinib concentration in various tissues at different time points.  Figure 4 reveals that lorlatinib was rapidly and widely distributed in different tissues. In each tissue, the maximum concentration of lorlatinib appeared at 0.5 h. At this time point, the highest concentration was found in the liver (3,153.93 ng/100 mg), followed by the stomach (2,159.92 ng/100 mg), kidney (548.83 ng/100 mg), small intestine (540.64 ng/100 mg), heart (367.31 ng/100 mg), lung (309.39 ng/100 mg), large intestine (289.85 ng/100 mg), spleen (246.81 ng/100 mg), and brain (185.03 ng/100 mg). The first pass effect of oral administration might be the main cause of high distribution in the liver. The high concentration in the stomach and small intestines suggested that lorlatinib was primarily absorbed by the stomach and small intestines. Although lorlatinib, a third-generation ALK inhibitor, is specifically designed to penetrate the blood-brain barrier, there was no expected high concentration of lorlatinib in the brain. In contrast, there was a higher distribution of lorlatinib in muscle tissues, such as heart, indicating that the lipophilicity of lorlatinib was not high enough to help the drug cross the blood-brain barrier. The kidneys had high concentration of lorlatinib, implying that lorlatinib might be excreted by the kidneys. High concentration of lorlatinib in the liver could be probably attributed to a slow metabolism in the liver, leading to a high lorlatinib concentration in the liver.

## 4. Conclusions

In the present study, we established a simple and rapid detection method and validated such method for lorlatinib determination in blood and tissue samples of mice. The pharmacokinetic study and tissue distribution were successfully investigated using this method. The high concentration of lorlatinib in the heart suggested that the lipophilicity of the drug was not as high as expected, resulting in low concentration in brain, even though lorlatinib is designed to penetrate the blood-brain barrier. High concentration of lorlatinib in the kidneys and livers implied that the kidney was the primary organ to eliminate lorlatinib, and metabolism in the liver was slow. The results of the pharmacokinetic study and tissue distribution provided valuable insights into the establishment of physiologically based pharmacokinetic model and its further evaluation of drug concentration in the brain after lorlatinib administration.

## Figures and Tables

**Table 1:** The mass spectrometric parameters of lorlatinib and IS.

No.	Compound	Molecular ion (*m/z*)	Fragmentation ion (*m/z*)	Cone voltage (V)	Collision energy (V)
1	Lorlatinib	407.28 (quantitative)	228.09	50	22
		407.28 (qualitative)	180.08	50	22
2	Afatinib-d6	492.10	371.05	58	28

**Table 2:** Linearity of calibration curve.

Sample	Linear equation	*R* ^2^	Linear ranges (ng/mL)
Serum	*y* = 0.023707 ∗ *x* + 0.0155831	0.997851	5.0–1000.00
Lung	*y* = 0.0195495 ∗ *x* + 0.0138712	0.997790	5.00–500.00
Heart	*y* = 0.0213465 ∗ *x* + 0.0092521	0.997465	5.00–500.00
Liver	*y* = 0.0186958 ∗ *x* + 0.0147887	0.998566	5.00–500.00
Stomach	*y* = 0.0204515 ∗ *x* + 0.0166741	0.999105	5.00–500.00
Spleen	*y* = 0.0202389 ∗ *x* + 0.0140355	0.995428	5.00–500.00
Brain	*y* = 0.0204129 ∗ *x* + 0.00546792	0.999465	5.00–500.00
Kidney	*y* = 0.019028 ∗ *x* + 0.00742472	0.999837	5.00–500.00
Large intestine	*y* = 0.0210146 ∗ *x* + 0.00305651	0.998751	5.00–500.00
Small intestine	*y* = 0.0223395 ∗ *x* + 0.0109578	0.997797	5.00–500.00

**Table 3:** Accuracy and precision for samples (within-day *n*=6; between-day *n*=18).

Samples	Concentration spiked (ng/ml)	Within-day accuracy (%)	Within-day precision (%)	Between-day accuracy (%)	Between-day precision (%)
Serum	5.00	3.87	2.99	10.17	10.46
	10.00	−1.92	5.04	0.68	7.60
	100.00	2.33	5.01	0.52	7.01
	800.00	2.01	4.06	−0.39	7.18

Lung	5.00	4.83	4.57	0.41	4.86
	10.00	8.35	7.23	6.34	4.82
	100.00	3.62	4.45	3.70	3.38
	400.00	1.96	4.61	2.56	3.36

Heart	5.00	−3.53	6.15	−5.31	5.13
	10.00	7.78	5.51	6.77	4.27
	100.00	6.31	4.79	8.20	5.54
	400.00	5.12	4.70	7.68	4.19

Liver	5.00	1.07	4.07	0.07	7.91
	10.00	4.23	4.77	8.84	7.11
	100.00	6.93	2.50	8.84	4.36
	400.00	4.91	6.14	5.06	4.26

Stomach	5.00	−12.17	5.13	−6.02	8.52
	10.00	0.23	8.24	2.46	5.04
	100.00	1.29	4.01	−0.16	4.03
	400.00	−3.37	3.25	−2.98	3.53

Spleen	5.00	−5.27	6.96	1.22	6.71
	10.00	6.47	2.86	7.94	4.52
	100.00	3.66	7.83	2.74	6.42
	400.00	5.12	3.11	1.21	5.18

Brain	5.00	−1.33	4.04	−0.61	5.12
	10.00	6.50	4.33	0.68	10.38
	100.00	6.07	7.66	0.35	10.98
	400.00	5.50	1.69	−0.42	8.09

Kidney	5.00	−1.73	4.42	−0.82	8.38
	10.00	2.27	3.20	5.91	5.04
	100.00	−3.31	5.43	1.50	5.24
	400.00	−4.68	5.53	1.26	6.64

Large intestine	5.00	−7.47	6.75	−0.94	19.73
	10.00	−0.72	6.82	−3.37	9.60
	100.00	3.20	3.70	−6.42	8.89
	400.00	0.12	2.78	−4.90	8.00

Small intestine	5.00	2.5	5.26	−7.1	9.26
	10.00	3.03	2.38	−0.48	7.79
	100.00	−1.13	3.42	−0.89	3.58
	400.00	−3.43	6.30	−1.78	4.35

**Table 4:** Recovery and matrix effect (*n*=6).

Samples	Concentration spiked (ng/ml)	Matrix effect (%)	Recovery (%)
Serum	10	108.9	102.0
	100	110.6	103.8
	800	105.7	101.4

Lung	10	90.4	99.5
	100	85.0	103.5
	500	85.3	98.5

Heart	10	93.3	104.4
	100	88.2	101.4
	500	86.8	99.1

Liver	10	86.8	109.6
	100	88.2	102.5
	500	91.1	98.1

Stomach	10	91.4	110.8
	100	95.5	108.3
	500	89.4	102.7

Spleen	10	89.0	112.1
	100	99.0	102.0
	500	91.0	108.1

Brain	10	91.3	115.0
	100	97.5	101.2
	500	96.0	91.4

Kidney	10	84.2	112.3
	100	89.5	107.2
	500	88.7	105.5

Large intestine	10	89.4	105.4
	100	84.5	99.0
	500	84.6	103.1

Small intestine	10	97.7	93.9
	100	97.0	93.2
	500	101.5	96.8

**Table 5:** Pharmacokinetic parameters of lorlatinib in mice after oral administration at a dose of 10 mg/kg (*n*=8).

Parameters	Unit	Mean	SD	RSD (%)
AUC(0–∞)	ug/L ∗ h	16208.177	1720.36	10.61
AUMC(0–∞)	h ∗ h ∗ ug/L	78840.738	10257.22	13.01
*t*1/2*z*	h	3.261	0.168	5.17
*T*_max_	h	0.625	0.231	37.03
*C*_max_	ug/L	2705.683	539.779	19.95

## Data Availability

The data used to support the findings of this study are available from the corresponding author upon request.

## Abbreviations

AUC: Area under the curve
AUMC: Area under the moment curve
ESI: Electron spray ionization
FDA: Food and Drug Administration
HPLC: High-performance liquid chromatography
IS: Internal standard
LLOQ: Lower limit of quantification
LC-MS/MS: Liquid chromatography-tandem mass spectrometry
MRT: Mean residence time
NSCLC: Non-small cell lung cancer
QC: Quality control
TKIs: Tyrosine kinase inhibitors.
